# Supplementary material for: Behavioral, health- related and demographic risk factors of death in floods: A case-control study
Source: PLoS One. 2021 Dec 31;16(12):e0262005. doi: 10.1371/journal.pone.0262005 (PMC8719708; doi:10.1371/journal.pone.0262005)
Supplement: Attachment — Submitted filename: PONE-D-21-06605_reviewer (1).pdf [file pone.0262005.s001.pdf]

PLOS ONE

How behavior influence flood deaths? A case control study

--Manuscript Draft--

**Manuscript Number:**	PONE-D-21-06605
**Article Type:**	Research Article
**Full Title:**	How behavior influence flood deaths? A case control study
**Short Title:**	behavior influence flood deaths
**Corresponding Author:**	Abbas Ostadtaghizadeh, Ph.D.<br>Tehran University of Medical Sciences<br>Tehran, Tehran IRAN, ISLAMIC REPUBLIC OF
**Keywords:**	Flood Death, Behavioral, Health Status, Demographic, Iran
**Abstract:**	Aims<br>During the first half of 2019, many provinces of Iran were affected by floods, which claimed the lives of 82 people. The present study aimed to investigate the behavioral and demographic risk factors in the deaths due to flood.<br>Methods<br>We measured the odds ratio and investigated the contribution and significance of the factors in relation to mortality. This case-control study was conducted in the cities affected by flood in Iran. Data were collected on the flood victims using a questionnaire and survey. In total, 77 subjects completed the survey in the case group, and 310 subjects completed the survey in the control group.<br>Results<br>The findings indicated that factors such as the age of less than 18 years, low literacy, being trapped in buildings/cars, and risky behaviors increased the risk of flood deaths. Regarding the behavioral factors, perceived/real swimming skills increased the risk of flood deaths although it may seem paradoxical. On the other hand, skills and abilities such as evacuation, requesting help, and escape decreased the risk of flood deaths.<br>Conclusions<br>According to the results, the adoption of support strategies, protecting vulnerable groups, and improving the socioeconomic status of flood-prone areas could prevent and reduce the risk of flood deaths.
**Order of Authors:**	Arezoo Yari
	Homa Yousefi- Khoshsabeghe2
	Yadolah Zarezadeh
	Ali Ardalan
	Mohsen Soufi Boubakran
	Mohamad Esmaeil Motlagh
	Abbas Ostadtaghizadeh, Ph.D.
**Additional Information:**	
**Question**	**Response**
**Financial Disclosure**<br><br>Enter a financial disclosure statement that describes the sources of funding for the work included in this submission. Review the submission guidelines for detailed requirements. View published research articles from *PLOS ONE* for specific examples.	This study (no: IR.MUK.REC.1398.223.) was conducted with the financial support of the Deputy for Health at Ministry of Health and Medical Education (MOHE) in IRAN. The funding body did not have any other roles in the study design, collection, analysis, and interpretation of data and in writing the manuscript.

Powered by Editorial Manager® and ProduXion Manager® from Aries Systems Corporation

This statement is required for submission and **will appear in the published article** if the submission is accepted. Please make sure it is accurate.<br><br>**Unfunded studies**<br>Enter: *The author(s) received no specific funding for this work.*<br><br>**Funded studies**<br>Enter a statement with the following details:<br>• Initials of the authors who received each award<br>• Grant numbers awarded to each author<br>• The full name of each funder<br>• URL of each funder website<br>• Did the sponsors or funders play any role in the study design, data collection and analysis, decision to publish, or preparation of the manuscript?<br>• **NO** - Include this sentence at the end of your statement: *The funders had no role in study design, data collection and analysis, decision to publish, or preparation of the manuscript.*<br>• **YES** - Specify the role(s) played.<br><br>* typeset	
**Competing Interests**<br><br>Use the instructions below to enter a competing interest statement for this submission. On behalf of all authors, disclose any competing interests that could be perceived to bias this work—acknowledging all financial support and any other relevant financial or non-financial competing interests.<br><br>This statement is required for submission and **will appear in the published article** if the submission is accepted. Please make sure it is accurate and that any funding sources listed in your Funding Information later in the submission form are also declared in your Financial Disclosure statement.<br><br>View published research articles from *PLOS ONE* for specific examples.	The authors have declared that no competing interests exist.

Powered by Editorial Manager® and ProduXion Manager® from Aries Systems Corporation

**NO authors have competing interests**<br><br>Enter: *The authors have declared that no competing interests exist.*<br><br>**Authors with competing interests**<br><br>Enter competing interest details beginning with this statement:<br><br>*I have read the journal's policy and the authors of this manuscript have the following competing interests: [insert competing interests here]*<br><br>* typeset	
**Ethics Statement**<br><br>Enter an ethics statement for this submission. This statement is required if the study involved:<br><br>• Human participants<br>• Human specimens or tissue<br>• Vertebrate animals or cephalopods<br>• Vertebrate embryos or tissues<br>• Field research<br><br>Write "N/A" if the submission does not require an ethics statement.<br><br>General guidance is provided below. Consult the submission guidelines for detailed instructions. **Make sure that all information entered here is included in the Methods section of the manuscript.**	The study protocol was approved by the Institutional Review Board (IRB) of Kurdistan University of Medical Sciences (ethics code: IR.MUK.REC.1398.223); the IRB follows the stipulated clauses of the Declaration of Helsinki.

Powered by Editorial Manager® and ProduXion Manager® from Aries Systems Corporation

**Format for specific study types**<br><br>**Human Subject Research (involving human participants and/or tissue)**<br>• Give the name of the institutional review board or ethics committee that approved the study<br>• Include the approval number and/or a statement indicating approval of this research<br>• Indicate the form of consent obtained (written/oral) or the reason that consent was not obtained (e.g. the data were analyzed anonymously)<br><br>**Animal Research (involving vertebrate animals, embryos or tissues)**<br>• Provide the name of the Institutional Animal Care and Use Committee (IACUC) or other relevant ethics board that reviewed the study protocol, and indicate whether they approved this research or granted a formal waiver of ethical approval<br>• Include an approval number if one was obtained<br>• If the study involved *non-human primates*, add *additional details* about animal welfare and steps taken to ameliorate suffering<br>• If anesthesia, euthanasia, or any kind of animal sacrifice is part of the study, include briefly which substances and/or methods were applied<br><br>**Field Research**<br><br>Include the following details if this study involves the collection of plant, animal, or other materials from a natural setting:<br>• Field permit number<br>• Name of the institution or relevant body that granted permission	
**Data Availability**<br><br>Authors are required to make all data underlying the findings described fully available, without restriction, and from the time of publication. PLOS allows rare exceptions to address legal and ethical concerns. See the PLOS Data Policy and FAQ for detailed information.	No - some restrictions will apply

A Data Availability Statement describing where the data can be found is required at submission. Your answers to this question constitute the Data Availability Statement and **will be published in the article**, if accepted.<br><br>**Important:** Stating 'data available on request from the author' is not sufficient. If your data are only available upon request, select 'No' for the first question and explain your exceptional situation in the text box.<br><br>Do the authors confirm that all data underlying the findings described in their manuscript are fully available without restriction?	
**Describe where the data may be found in full sentences. If you are copying our sample text, replace any instances of XXX with the appropriate details.**<br><br>• If the data are **held or will be held in a public repository**, include URLs, accession numbers or DOIs. If this information will only be available after acceptance, indicate this by ticking the box below. For example: *All XXX files are available from the XXX database (accession number(s) XXX, XXX.*).<br>• If the data are all contained **within the manuscript and/or Supporting Information files**, enter the following: *All relevant data are within the manuscript and its Supporting Information files.*<br>• If neither of these applies but you are able to provide **details of access elsewhere**, with or without limitations, please do so. For example:<br><br>*Data cannot be shared publicly because of [XXX]. Data are available from the XXX Institutional Data Access / Ethics Committee (contact via XXX) for researchers who meet the criteria for access to confidential data.*<br><br>*The data underlying the results presented in the study are available from (include the name of the third party*	Data cannot be shared publicly because of the confidentiality of the data and observance of ethical principles.

and contact information or URL).<br>• This text is appropriate if the data are owned by a third party and authors do not have permission to share the data.<br><br>* typeset	
Additional data availability information:	

Manuscript
Click here to access/download;Manuscript;Manuscript.docx

How behavior influence flood deaths? A case control study

Arezoo Yari[1], Homa Yousefi- Khoshsabeghe[2], Yadolah Zarezadeh[1], Ali Ardalan[2], Mohsen Soufi Boubakran[3], Abbas Ostadtaghizadeh*[1], Mohamad Esmaeil Motlagh[4]*

1. Social Determinants of Health Research Center, Research Institute for Health Development, Kurdistan University of Medica Sciences, Sanandaj, Iran.

2. Department of Health in Emergencies and Disasters, School of Public Health, Tehran University of Medical Sciences, Tehran,I.R.IRAN.

3. Department of Mechanical Engineering, Urmia University, Urmia, Iran

4. Department of Pediatrics, Ahvaz Jundishapur, University of Medical Sciences, Ahvaz, Iran

***First Corresponding Author:** Abbas Ostadtaghizadeh
Address: Poorsina Ave, Department of Health in Emergencies and Disasters, School of Public Health, Tehran University of Medical Sciences, Tehran, Iran
Post Code: 14177-43578
Telephon: 00989123909011
Fax:- 0098- 021- 88991108
Email: ostadtaghizadeh@gmail.com

****Second Corresponding Author:** Mohamad Esmaeil Motlagh
Address: Esfand Ave, Department of Pediatrics, Ahvaz Jundishapur, University of Medical Sciences, Ahvaz, Iran
Post Code: 61357-15794
Telephone: 00989122995592
Fax: 0098- 061- 33367543
Email: Dr.motlagh.ms@gmail.com

Behavioral and Demographic Risk Factors of Death in Floods: A Case-Control Study

Abstract

I am not sure whether the Journal guideline follows structured way of abstract (aim,methods,results..conclusion..)

Aims

During the first half of 2019, many provinces of Iran were affected by floods, which claimed the lives of 82 people. The present study aimed to investigate the behavioral and demographic risk factors in the deaths due to flood.

Methods

We measured the odds ratio and investigated the contribution and significance of the factors in relation to mortality. This case-control study was conducted in the cities affected by flood in Iran. Data were collected on the flood victims using a questionnaire and survey. In total, 77 subjects completed the survey in the case group, and 310 subjects completed the survey in the control group.

What does mean questionnaire and survey@line 46? Subject is not also appropriate way of explanation. Use standard research language.

Results

The findings indicated that factors such as the age of less than 18 years, low literacy, being trapped in buildings/cars, and risky behaviors increased the risk of flood deaths. Regarding the behavioral factors, perceived/real swimming skills increased the risk of flood deaths although it may seem paradoxical. On the other hand, skills and abilities such as evacuation, requesting help, and escape decreased the risk of flood deaths.

You should have justification for this padoxical results either literature driven or data driven justification

Conclusions

Conclusion and recommendation is two different things.

According to the results, the adoption of support strategies, protecting vulnerable groups, and improving the socioeconomic status of flood-prone areas could prevent and reduce the risk of flood deaths.

Keywords: Flood Death, Behavioral, Health Status, Demographic, Iran

correct as Demographic factor

Introduction

The 2018 World Disasters Report indicated that 1,522 floods have occurred in the world within the past decade (2008-2017). Statistics suggest that approximately 37% of the two billion people affected by natural disasters in this period were impacted by floods, and 50,312 deaths have been recorded due to floods [1]. Iran is one of the world's most flood-prone countries [2]. In the first half of 2019; heavy rains flooded different regions of the country [3], and this unprecedented disaster caused huge damages, such as death, damage and destruction of buildings and vital infrastructures, burden on the healthcare infrastructure, and destruction and damage of properties and assets (e.g., livestock and agricultural lands) [4]. Furthermore, about 10 million people in more than 2,000 cities and towns across the country have been affected by floods (Figures 1 & 2), and more than half a million were evacuated permanently or temporarily from their homes [3]. Among the affected provinces in Iran, 200 towns and 4,304 villages were damaged totally or partially. Furthermore, the complete destruction of 6,000 urban and rural housing units and damage to more than 75,000 urban and rural housing units were recorded as the detriments caused to properties and assets by floods [4].

repetition of ideas

Which literature ? either cite the reference or rewrite

Floods are a major cause of death among various natural disasters [5]. According to the literature, the causes of flood death could be classified into two general categories, which are the immediate causes of death, such as drowning [6], physical trauma, heart attack, fire, electrocution, over-exertion, and shock, and underlying factors, including hazard-related factors, and individual, environmental, socioeconomic, and managerial factors [7]. The degree of individual vulnerability, health status and behavior of individuals in the areas affected by floods [8-11], and their response to floods [12,13] are also classified as the underlying factors of flood

deaths. In fact, flood deaths result from long-term attitudes, behaviors, decision-making, and community actions [14].

A high percentage of flood deaths in Europe are attributed to high-risk behaviors [13,15]. In 2002, the World Health Organization (WHO) reported that approximately 40% of the health effects of floods in Europe are associated with high-risk behaviors [16]. In addition, several of these high-risk behaviors during floods are unnecessary [11,17], such as using vehicles in the United States during floods, which result in flood deaths [15,18]. In 2002, Egli also stated that in Switzerland, 40% of the 67 flood deaths during 1972-2001 occurred due to the inappropriate behaviors of the victims [19]. A combination of risk factors and individual vulnerabilities has been shown to cause death in floods [6,13], and the response of people to floods plays a key role in determining the mortality and morbidity of floods [17].

Awareness, behaviors, decision-making, resilience, problem-solving, skills in dealing with floods, and precautionary measures could help reduce flood deaths. Human behavior and decision-making during natural disasters is a complex process. It is the result of a combination of environmental factors and social processes with individual factors and abilities [20]. Prior experience with flood, trust in public protection (government's protective measures), and excessive emotions affect people's behavior during floods. Notably, decisions to respond to threats, the ability to respond, and the evaluation of the costs and effectiveness of methods affect the decisions of individuals on their response to flood risks. Furthermore, beliefs about the effectiveness of preventive behaviors influence precaution measures, and higher risk perception seems to be associated with better self-protection and preventive measures during floods [21].

Which characteristics? describe as Socio demographic or else..

In addition to perceptual factors, characteristics such as age, gender, marital status, education level, and attitude toward swimming skills may affect people's risk

acceptance and risky behaviors during floods [22]. Depending on the features of the region's population, the mortality rate may increase during floods, and the demographic features of the flood-prone area may expose the youth or vulnerable groups (e.g., women, the elderly) to the higher risk of death [17]. Subsequently, protective measures for hazards and adopting protective behaviors should be promoted among the elderly [23,24]. However, Diakakis et al in 2018 claimed that although the elderly tends to assess their knowledge of floods and flood protection measures more commonly than the youth, age and protective measures have no significant association in this regard [25].

Women and elderly(Use appropriate conjunction like and ..

Flood management efficiency is of great importance considering the adverse effects of this natural disaster on the loss of life and economic losses [26]. Death is a serious and irreversible effect following floods [27], and compared to other natural disasters, the frequency of deaths due to floods has been reported to be noticeably higher [28-30]. Moreover, the flood death trend has been reported to be on the rise across the world [31,32]. The increased rate of floods and the resulting deaths are more significant in Asia, as well as developing and underdeveloped countries [7]. One of the primary goals of the Sendai framework for disaster risk reduction in 2015-2030 is to reduce deaths in the populations affected by natural hazards. Henceforth, the implementation of risk management policies and appropriate interventions to reduce flood deaths necessitates the assessment of the influential factors in flood deaths [26].

Considering the impact of behavior, health status, and demographic factors on the flood deaths and floods in Iran in 2019 and in line with the main objectives of the Sendai framework, the present study aimed to determine the behaviors, health status, and demographic factors associated with flood deaths and evaluate the significant correlations of the factors related to flood deaths and the significance of their effects.

The required data were collected and compared on the individual and behavioral factors of recent flood deaths from the families of the victims and survivors by conducting a survey. Another objective of the research was to determine the optimal preventive strategies based on the most significant factors in this regard.

Methods

This case-control study aimed to investigate the flood deaths that occurred within the first six months of 2019 in Iran based on the behaviors, health status, and demographic factors affecting the flood deaths, which were assessed retrospectively.

What was your justification to take only these limited variables? The paper don't have any theoretical framework as the base for the study

Cases and Controls

In the current research, the victims who had died due to direct contact with the floods in 2019 were classified as the case group. If the individual facing the floods had died due to other causes than flood, they would not be considered as cases. The control group included the survivors or living individuals in the flooded areas during the floods that caused the deaths of the flood victims, including the residents of the flooded areas in the recent incidents. To select the controls via population-based sampling, we randomly selected the control samples from the neighbors of the flood victims based on family health dossier number in the Comprehensive Health Services and Health Centers (10 numbers above and below).

not clear. Rewrite the language

Study Design and Setting

In 2019, several floods occurred in Iran, in which 2,193 people were injured, and 82 died in the affected provinces (Figures 1 & 2). The study areas in the present study were different cities across Iran that had been affected by the deaths occurring in the successive floods in 2019. In Figures 1 and 2, the provinces that were not affected by the floods are shown in gray, and those affected by the floods are shown in

different shades of orange and red. Increased color intensity in the affected provinces (tending to red) indicated the higher rates of deaths and injuries.

Sample Size and Sampling

In the present study, almost all the Iranian records of flood deaths were reviewed, including the records of the Disaster Management Organization, the Governorate, the Ministry of Energy, the Ministry of Health (Public Health and Emergency Operation Center), and Forensic Medicine. In addition, we reviewed the data to ensure the proper confirmation of the recorded death rates, which enables us to attain the demographic data of the flood victims as well, including age, gender, geographical location, home address, flood time, and phone number of the closest family member/other persons. In total, 86 cases of flood deaths were reviewed, which had been recorded in various sources of the floods occurring in Iran during 17th March-29th May 2019.

Which Governorate? use appropriate language

Based on the mentioned definition of flood death, only 82 deaths could be identified as four cases had died due to other reasons coinciding with the floods. We investigated the behaviors, health status, and demographic causes of these 82 deaths. During the investigation, the address and phone numbers of two flood victims' relatives were false, and follow-up was not possible. In addition, three relatives of the flood victims were unwilling to partake in the study. Finally, the study was performed using the data of 77 flood victims who had died in Iran's floods in 2019. Approximately 93.9% of the relatives of the flood victims participated in the study.

This retrospective study was conducted with a case-control design and started three months after the floods occurring in Iran after 29th May, 2019. Therefore, the probability of recall bias was low, and the strength of the study was increased by considering the ratio of the controls to the cases to be 4:1. In other words, four controls were selected per each case of death.

Variable Measurements

Still there is language problem.
The investigated study doesn't make sense for the academia

The investigated study outcome was flood deaths, which pertained to the deaths caused by floods and defined as the deaths in direct association with the floods or a fatality that would not have occurred without a flood. In addition, the health authorities provided a list of the deaths in direct association with the floods, with the exposure factors mainly reported to be the demographic/behavioral factors and health status of the individuals, which could increase or decrease the risk of flood.

The individual characteristics included age, gender, education level, occupation status, number of the household members, place of residence (by province), place of residence (by city/village), ethnicity, and income and residence status (indigenous/non-indigenous). In addition, the behavioral and health states were examined in association with abilities and skills, general health, alcohol/drug consumption, prior experience, physical conditions, area recognition, timely escape, attention to safety and danger limits, attention to meteorological warnings, situation, and state during the accident.

Data collection tool was a reliable and valid questionnaire, which was developed in one of the dissertations of Health in Emergencies and Disasters Department of Tehran University of Medical Sciences entitled the "Questionnaire of the Factors Affecting Flood Deaths in Iran"[33]. The reliability of the scientific indices of the questionnaire items was evaluated psychometrically in the doctorate dissertation. In addition, the empirical validity of the instrument was assessed by factorial analysis using the Kaiser-Meyer- Olkin (KMO) test. In general, the KMO of higher than 0.6-0.7 in the exploratory factor analysis process indicates the suitability of the variables' number and sampling adequacy [34]. In the current research, the KMO coefficient of the questionnaire was higher than 0.6-0.7 in all the constructs, and Bartlett's test was also considered significant. The reliability or internal consistency of the

not clear

Which current research?

inappropriate expression

questionnaire was also evaluated based on Cronbach's alpha, which was within the range of 0.75-0.91, demonstrating favorable/excellent internal consistency [34]. In addition, the internal consistency of the questionnaire was confirmed at the Cronbach's alpha coefficient of 0.92. The instrument was used to measure the influential factors in the flood deaths by 33 items and seven constructs; however, we only presented the demographic, behavioral, and health factors affecting the flood deaths in Iran's floods in 2019.

Surveyer ? Please use either data collector or enumerator

The surveyors were trained to help the participants respond to the survey questions and also provided the necessary explanations. The survey method was the same for the case and control group. To obtain the case data, the next-of-kin of the flood victims was asked to participate in the study, and the participants were preferably a member of the flood victim’s family with a higher education level and willingness to partake.

Matching

The is not self explanatory. What is Matching?

In the current research, the group-matching method was applied to facilitate the implementation of the survey and individual matching limitations. Frequency matching was also performed for the variable of the areas affected by the floods, and the control samples were selected as a random sample of the affected population. Furthermore, binary logistic regression analysis was used to ensure the thorough control of the confounding variables as it provided the odds ratio (OR) for controlling multiple confounders. The ORs were documented as the adjusted OR, which should be adjusted for other covariates (e.g., confounders) [35].

Statistical Analysis

Descriptive and analytical statistics were used to evaluate and measure the variables by type and category of variables. The analytical statistics determined the

You should use appropriate place and appropriate description of variables. Avoid the bracket description @256.

correlations between the demographic variables, health status, and behavioral factors (independent variables) with the flood deaths (dependent variable) by Chi-square or Fisher's exact test based on the OR. In these tests, we only determined the presence or absence of correlations between the behavioral factors, health status, and demographic variables with the flood deaths. In the binary logistic regression analysis, the effects of these factors on the flood deaths were investigated as well. Moreover, binary logistic regression analysis was performed for all the significant variables in Chi-square or Fisher's exact test, and the effects of confounders were also controlled by the binary logistic regression analysis.

Ethical Considerations

This is not health study...check the journal guideline

The interviews were conducted with the participants' consent. After explaining the objectives of the study to the participants, they were assured of the confidentiality of the data and observance of ethical principles. The participants were also allowed to withdraw from the study at any stage or not answer some of the survey questions. The time and place of the interviews were determined by the participants so as to make them feel comfortable during the interviews. The study protocol was approved by the Institutional Review Board (IRB) of Kurdistan University of Medical Sciences (ethics code: IR.MUK.REC.1398.223); the IRB follows the stipulated clauses of the Declaration of Helsinki.

Results

What was your justification to increase the control group?

Descriptive and Analytical Results

In total, 387 participants were enrolled in the study, including 77 cases and 310 controls. The descriptive and simple analytical results of Chi-square or Fisher's exact test have been presented in the following sections, including demographic variables, behavioral factors, and health status.

Demographic Variables

The mean age of the participants was 38.8±15.14 years, and the male-to-female ratio was 3.83. In total, 23.3% of the participants were university graduates, 26.72% had a high school diploma, and 22.4% were illiterate. In terms of the residence, 50.4% of the participants were urban, and 49.6% were rural. In addition, 24.6% of the participants were self-employed, and 18.1% were employees. Notably, 84.5% of the participants lived in the flooded areas. The analytical results regarding the correlations between the behavioral and skill characteristics of the victims of flood death based on Chi-square or Fisher's exact test indicated that age, education level, and occupation status were significantly associated with flood death (P<0.005) (Table 1).

Category of demographic and other variables should have justification

Table 1. Association between demographic factors and flood death in Iran floods in 2019, (n=387)

Demographic Factors	Case(n=77), n (%)	Control(n=310), n (%)	Test Result	(P value) OR (95 % CI)
**Sex**				
**Female**	21(27.3)	54(17.4)	OR = 0.563	0.239-1.079
**Male**	56(72.7)	256(82.6)		
**Age Group (Year)**				
**<18**	18(23.4)	10(3.2)	X^2 =27.52	0.000
**18-28**	11(14.3)	40(12.9)		
**28-38**	12(15.6)	100(32.3)		
**38-48**	16(20.8)	82(26.5)		
**48-58**	12(15.6)	44(14.2)		
**>58**	8(10.4)	34(11.0)		
**Education**				
**Illiterate**	27(35.1)	50(16.2)	X^2 =17.99	0.003
**Primary**	10(13.0)	18(5.8)		
**Elementary**	11(14.3)	12(3.8)		
**High school**	6(7.8)	44(14.2)		
**Diploma**	10(13.0)	104(33.5)		
**University**	13(16.8)	82(26.5)		
**Occupation**				
**Driver**	4(5.2)	22(7.1)	Exact= 29.39	0.000
**rancher or farmer**	13(16.9)	40(12.9)		
**Student (school)**	14(18.2)	6(1.9)		
**Student (university)**	0(0.0)	6(1.9)		
**Employee**	10(13.0)	64(20.6)		

Table 1. Association between demographic factors and flood death in Iran floods in 2019, (n=387)				
**Demographic Factors**	Case(n=77), n (%)	Control(n=310), n (%)	Test Result	(P value) OR (95 % CI)
**Self- Employment**	11(14.3)	92(29.7)		
**Unemployed**	5(6.5)	10(3.2)		
**Housewife**	14(18.2)	54(17.4)		
**Retired**	6(7.8)	16(5.2)		
**Living Place**				
City	45(58.4)	144(41.6)	OR= 1.621	0.933-2.816
Village	32(46.5)	166(53.5)		
**Residency situation**				
Resident	65(84.4)	262(84.5)	OR= 0.992	0.467-2.109
Non-Resident	12(15.6)	48(15.5)		

Behavioral Factors

The results of the descriptive analysis regarding the behavioral factors of the participants indicated that 98.7% had not consumed alcohol at the time of the floods. Additionally, 74.1% of the participants had not experienced floods before, and area recognition was 52.1% above average. Attention to safety and danger limits in 51.3% and attention to meteorological warnings in 45.3% of the participants were moderate. However, these rates were higher in the control group compared to the case group.

During the floods, 42.2% of the participants had high self-esteem and 'heroic behaviors', 5.2% had risky behaviors, 3.9% of the participants had walked into or entered the floodwaters (flooded river, bridge or road), and 8.2% had been trapped in a building or a vehicle. Regarding the behavioral factors based on abilities and skills, 32.7% of the participants were able to evacuate, and 9.5% could swim; on the other hand, 61% of the subjects in the case group had no such skills or abilities. The subjects in the control group had one or more skills, and the lack of skills or abilities was not observed in this group. Furthermore, the ability to request help, escape, and scram was greater in the control group compared to the case group.

The analytical results of Chi-square or Fisher's exact test indicated significant correlations between flood death and timely escape, the observance of safety and danger limits, situation and state, attention to meteorological warnings, and type of abilities and skills (Table 2).

Table 2. Association between personal and behavioral factors and flood death in Iran floods in 2019, (n=387)

Personal and Behavioral Factors	Case(n=77), n (%)	Control(n=310), n (%)	Test Result	(P value) OR (95 % CI)
**Alcohol Consumption**				
Yes	1(1.3)	4(1.3)	OR = 1.007	0.090 -11.276
No	76(98.7)	306(98.7)		
**Having Flood Experience**				
Yes	17(22.1)	86(27.7)	OR = 0.738	0.388-1.404
No	60(77.9)	224(72.3)		
**Proper Escape Ability**				
Yes	16(20.8)	204(65.8)	OR = 0.505	0.265-0.960
No	61(79.2)	106(34.2)		
**Knowledge of the flooded area**				
Very High	30(39.0)	116(37.4)	X^2= 7.40	0.116
High	8(10.4)	50(16.1)		
Average	13(16.9)	82(26.5)		
Low	8(10.4)	16(5.2)		
Very Low	18(23.3)	46(14.8)		
**Attention to safety principles and settlement risk**				
Very High	16(20.8)	84(27.1)	X^2=12.61	0.013
High	8(10.4)	106(34.1)		
Average	31(40.3)	84(27.1)		
Low	10(13.0)	20(6.5)		
Very Low	12(15.6)	16(5.1)		
**Attention to meteorological warnings**				
Very High	20(26.0)	68(21.9)	X^2= 15.21	0.004
High	11(14.3)	80(25.8)		
Average	18(23.4)	110(35.5)		
Low	7(9.1)	8(2.6)		
Very Low	21(27.3)	44(14.2)		
**Individual statues at flood time**				
Self-confidence and heroism	50(64.9)	96(30.9)	X^2= 29.71	0.000

Table 2. Association between personal and behavioral factors and flood death in Iran floods in 2019, (n=387)				
Personal and Behavioral Factors	Case(n=77), n (%)	Control(n=310), n (%)	Test Result	(P value) OR (95 % CI)
Trapping *	9(11.7)	20(6.5)		
Risky behavior *	8(10.4)	8(2.6)		
Crossing *	9(11.7)	0(0.0)		
Fear and stress	1(1.3)	186(60.0)		
**Type of ability and skill**				
Swimming	15(19.5)	14(4.5)	X^2= 160.58	0.000
Evacuation	3(3.9)	146(47.1)		
Self- Rescue technique	2(2.6)	0(0.0)		
Decision making	1(1.3)	48(15.5)		
Help request	3(3.9)	58(18.7)		
Escape and get away	1(1.3)	44(14.2)		
Being multi-skilled	5(6.5)	0(0.0)		
Without skill and ability	47(61.0)	0(0.0)		

Health Status

The investigation of the health status indicated that 98.3% of the subjects had no specific disabilities or illnesses before the floods and used no special medications. Moreover, the analytical results regarding health status and flood death based on Chi-square or Fisher's exact test indicated no significant correlations between the health factors and flood death (Table 3).

Table 3. Association between health status and flood death in Iran floods in 2019, (n=387)				
Health Status	Case(n=77), n (%)	Control(n=310), n (%)	Odds Ratio	(95 % CI)
**Having disease**				
Yes	1(1.3)	4(1.3)	OR= 2.04	0.282-14.765
No	76(98.7)	306(98.7)		
**Having Disability**				
Yes	3(3.9)	2(0.6)	OR= 6.24	0.639- 61.044
No	74(96.1)	308(99.4)		
**Medication Consume**				
Yes	2(2.6)	4(1.3)	OR= 2.04	0.282- 14.765
No	75(97.4)	306(98.7)		

Binary Logistic Regression

Unnecessary description of variables@324.

The results of binary logistic regression analysis indicated that age and literacy level (dependent variables) were significantly correlated with flood death. For instance, the age of less than 18 years compared to the age of more than 58 years was observed to increase the risk of death significantly (OR=9.15; 95% CI: 3.12-11.97; P<0.034). Similarly, illiteracy level (OR=5.42; 95% CI: 2.13-10.32; P<0.02), low literacy (OR=3.03; 95% CI: 1.36-13.67; P<0.03), and intermediate literacy (OR=3.23; 95% CI: 3.26-17.94; P<0.04) also increased the risk of flood death compared to academic education (Table 4).

Avoid the SE,Wald...as you are not used in the description. use only relevant tests for your analysis Table 4

Table 4. Beta coefficients and test statistics of the variables used in the logistic regression equation

Variable	B	S. E	Wald	df	sig	Exp (B)	(95 % CI) for EXP(B)
**Age**							
<18	3.854	2.981	0.387	1	0.034	9.157	3.123-11.974
18-28	1.220	1.943	1.041	1	0.530	0.295	0.007-13.320
28-38	2.249	2.497	0.875	1	0.894	9.482	0.071-12.997
38-48	0.265	1.988	2.413	1	0.532	1.303	0.026-64.188
48-58	1.053	1.686	0.071	1	0.129	2.867	0.105-78.089
**Education**							
Illiterate	5.580	2.590	4.641	1	0.021	5.425	2.134-10.326
Primary	17.313	32.24	2.576	1	0.036	3.036	1.362-13.672
Elementary	6.026	2.309	0.211	1	0.049	3.234	3.264-17.948
High school	4.216	3.157	0.94	1	0.182	5.064	0.863-7.177
Diploma	2.965	2.463	0.000	1	0.229	6.593	0.984-6.440
**Occupation**							
Driver	2.697	2.419	1.243	1	0.265	6.235	0.001-7.718
rancher or farmer	3.383	1.784	3.596	1	0.058	9.034	0.001-1.120
Student (school)	4.245	3.202	1.775	1	0.185	5.061	0.372-65.354
Student (university)	-12.003	25.998	1.758	1	0.356	4.014	0.000-3.267
Employee	-3.320	2.494	0.000	1	0.183	21.036	0.000-4.798
Self- Employment	2.798	2.098	1.772	1	0.161	6.061	0.001-1.722
Unemployed	3.763	2.687	1.962	1	0.998	0.023	0.389-41.798
Retired	-8.315	27.88	0.000	1	0.219	4.028	2.689-69.491

Table 4. Beta coefficients and test statistics of the variables used in the logistic regression equation

Variable	B	S. E	Wald	df	sig	Exp (B)	(95 % CI) for EXP(B)
**Attention to safety principles and settlement risk**							
Very High	-5.432	3.120	2.493	1	0.016	3.159	2.004-8.307
High	-4.768	2.226	2.575	1	0.037	4.061	1.037-6.819
Average	-0.713	1.902	1.673	1	0.460	4.191	0.004-1.307
Low	-1.273	2.229	2.813	1	0.519	9.014	0.000-2.687
**Attention to meteorological warnings**							
Very High	-1.273	2.229	0.328	1	0.568	0.280	0.004-22.098
High	-4.768	2.226	0.078	1	0.032	0.008	0.000-0.666
Average	-0.713	1.902	1.340	1	0.708	0.490	0.012-20.393
Low	-5.432	3.120	0.161	1	0.082	0.004	0.000-1.982
**Individual statues at flood time**							
Self-confidence and heroism	6.297	3.351	0.545	1	0.060	54.313	0.764-38.635
Trapping *	10.081	4.214	2.585	1	0.017	23.612	6.182-15.369
*Risky behavior	22.480	28.783	0.012	1	0.022	37.926	12.348-23.377
*Crossing	21.182	92.136	1.515	1	0.024	19.865	8.651-19.349
**Type of ability and skill**							
Swimming	37.502	13.042	0.123	1	0.048	1.937	2.165-9.321
Evacuation	-43.986	13.0245	0.235	1	0.021	14.267	1.256-8.325
Self- Rescue technique	-3.227	27.951	0.023	1	0.561	7.046	0.265-123.569
Decision making	-37.803	42.327	0.0345	1	.048	26.315	2.984-15.371
Help request	-4.215	33.178	0.326	1	.027	39.261	1.348-10.258
Escape and get away	-30.038	13.669	0.017	1	0.017	25.063	2.639-13.951
Being multi-skilled	-45.046	15.694	0.125	1	0.997	14.621	0.436-36.327
**Proper Escape Ability**							
Yes	-2.631	1.233	0.087	1	0.033	13.888	1.239-15.636

Considering the significant correlation of the safety and danger limits (dependent variable) with the flood deaths, very high attention (OR=3.15; 95% CI: 2.04-8.30; P<0.016) and high attention (OR=4.06; 95% CI: 1.03-6.81; P<0.03) reduced the risk of flood deaths compared to inadequate attention to these principles. Individuals' situation and state during the accident (dependent variable) also had a significant

avoid it@333

association with the flood deaths, and trapping in a building or car (OR=23.61; 95% CI: 6.18-15.36; P<0.017) increased the risk of flood death. In addition, high-risk behaviors such as watching, tacking a picture, curiosity, relief efforts and rescue, retrieving stocks or properties, incaution (OR=37.92; 95% CI: 12.34-23.37; P<0.02), crossing flooded routes, rivers, roads, and bridges (OR=19.86; 95% CI: 8.65-19.34; P<02) increased the risk of flood deaths compared to the avoidance of high-risk behaviors due to stress or fear (Table 4).

The same problem@344

The type of skills and abilities (dependent variable) had a significant correlation with the flood deaths; paradoxically, perceived or real swimming skills (OR=1.93; 95% CI: 2.16-9.32; P<0.04) increased the risk of flood death compared to the lack of such skills and abilities. On the other hand, evacuation abilities (OR=14.26; 95% CI: 1.25-8.32; P<0.02), decision-making skills (OR=26.31; 95% CI: 2.98-15.37; P<0.04), ability to request help (OR=39.26; 95% CI: 1.34-10.25; P<0.02), and ability to escape (OR=25.06; 95% CI: 2.63-13.95; P<0.01) decreased the risk of flood death compared to the lack of such skills and abilities. In addition, escape at the time of a flood time (dependent variable) had a significant correlation with the flood deaths, while timely escape during the floods (OR=13.88; 95% CI: 1.23-15.63; P<0.03) reduced the risk of flood death compared lack thereof (Table 4).

Discussion

The language is poor

The present study aimed to determine the correlation between the behavioral factors, health status, and demographic variables with the flood deaths occurring in Iran's floods in 2019. This was descriptive and analytical study, which also compared these factors between the case and control groups, and the effect and size of the associations were also measured and interpreted using binary logistic regression analysis. The comprehensive survey of the behavioral factors, health status, and demographic variables clarified whether the recognized risk factors were

significantly correlated with flood death. Furthermore, we attempted to assess the significance of the correlation and whether it increased or decreased the risk of flood death.

Age was observed to be an influential factor in the flood deaths in the present study, the effect of which has also been confirmed the previous studies [29,36]. The regression analysis in the current research indicated that the age of less than 18 years extremely increased the risk of flood deaths, which is consistent with several studies suggesting that young age is a risk factor for flood deaths [18,28,29,37]. Therefore, the improvement and promotion of the safety of flood-prone areas where the youth reside, such as educational places (e.g., schools and institutions) and recreational/sports complexes could reduce the risk of flood death. In addition, some studies have proposed that aging is a risk factor for flood deaths [14,28,38], and it is essential to support and implement appropriate measures to protect vulnerable populations, such as the youth and the elderly [31]. Moreover, families should be advised to plan for the support and protection of vulnerable age groups during floods [39].

Our findings indicated that low education levels were a risk factor for flood deaths, which is consistent with the study by Samir. The mentioned study denoted that increased education level could lead to higher awareness, and the type of employment could also reduce the risk of injuries, as well as flood death.[40] The impact of education on risk reduction is evident [41,42] and has been emphasized in risk reduction and disaster management policies, particularly for the promotion of the culture of resilience in various communities [43]. Therefore, it could be concluded that higher education levels in flood-prone areas may decrease population vulnerability and flood deaths. Considering the mentioned findings regarding the effects of age and education level on flood deaths, managers and decision-makers

should pay special attention to the education and training of the youth based on school learning materials, which may involve the incorporation of survival strategies into the curriculum or better informing of the prevention of flooding through lifestyle changes.

In the present study, attention to safety and danger limits decreased the risk of flood death. Consistently, some studies have shown that disregarding and underrating the risk increases the risk of flood death [13,30,44]. Consequently, the principles of safety and danger limits must be implemented in residential, educational, recreational, governmental, and non-governmental places, especially in densely populated areas. Furthermore, informing the public and attracting their attention should be attained by installing signs, banners, and posters, as well as through the media and by other appropriate methods to attract their attention to safety principles and danger limits. This is important because early warning and sending alerts in flood-prone areas may play a key role in the reduction of flood deaths [45].

According to the current research, high-risk behaviors such as staying in cars or buildings when the situation demanded otherwise increased the risk of flood deaths. In this regard, the results of binary logistic regression analysis indicated that trapping in a building or car increased the risk of flood death. Previous studies have also shown that being trapped at home/in a building could increase the risk of flood deaths [46,17]. However, Brazdova and Riha in 2014 stated that buildings that could protect people from the ruins of floods or the effects of water waves may diminish the risk of flood deaths, which implied the importance of standard buildings to protect people during floods [47]. Therefore, the standardization and strengthening of buildings in flood-prone areas are highly recommended. In these areas, buildings should be designed with proper evacuation operations and escape attempts in the case of trapping in the building during floods.

The results of the regression analysis indicated that high-risk behavior such as watching the flood or tacking pictures, being curious, relief efforts and rescue, and retrieving stocks or properties significantly increased the risk of death. Moreover, the analysis showed that crossing flooded routes, river, roads, and bridges was correlated with the higher risk of flood deaths. Several studies have proposed that relief efforts and rescue [13,48], entering floodwaters [12] or taking unnecessary risks [47] are among the important influential factors in flood deaths. Therefore, it could be inferred that high-risk behavior increase the risk of flood death and should be avoided altogether. It also seems that the level of awareness and perceptions toward flood risks are rather low in communities, and the adoption of strategies such as educational interventions, raising public awareness on floods [49,50], preventive measures, public awareness measures in the prevention phase [51], and increasing preparedness and response are strongly recommended.

According to the current research, skills and abilities affected flood-related mortality. Paradoxically, perceived or real swimming skills increased the risk of flood death. Several studies have also confirmed that the people who try to walk into/swim in floods are more likely to die [12,13]. Perceived or real skills of swimming may also increase self-esteem and courage in confronting floodwater, thereby increasing the risk of death due to flood because the named skills are insufficient to survive as the nature and behavior of water are dramatically different from pools or lakes. As such, proper training of communities in flood-prone areas regarding the dangers of swimming and walking in floods is paramount. It is also imperative to empower communities in flood-prone areas to improve their decision-making abilities and demand help in the due time during floods. These recommendations are consistent with the policies of disaster risk reduction programs based on education and training to propagate the culture of resilience in various

communities [52]. Public education and training for facing floods before and during floods is among the key principles of disaster risk management [13].

In the present study, the results of regression analysis indicated that the possession of abilities and skills such as evacuation, prompt decision-making, requesting help, escape, and scramming could significantly reduce the risk of flood deaths, which implied the high chance of survival depending on the endurance and ability of individuals to find an appropriate shelter [17].

The characteristics of the people affected by floods and their ability to respond and ensure the safety of themselves and their relatives during the flood time could determine their vulnerability to floods [53]. For instance, the people who can make the right decisions, ask for help, act/behave properly [52], and escape in a timely manner [8-10] are at a lower risk of flood death. Considering the notable impact of increasing the self-care knowledge of the residents of flood-prone areas on the reduction of the associated fatalities [54], it is imperative to train people, perform proper exercises, and improve the abilities and skills of the community members in order to reduce the fatalities and damages caused by floods.

Despite the immutability of individual factors, the adoption of support strategies, protection of vulnerable groups, and improvement of socioeconomic factors in flood-prone areas could effectively prevent and decrease the risk of flood deaths. In this regard, we propose several measures, including the adoption of harm reduction policies, improvement and strengthening of socioeconomic support, and promotion of inter-organizational and inter-sectoral cooperation. Furthermore, the optimization of disaster education systems, implementation of educational measures (especially behavioral and skill-based measures), and establishing and strengthening natural hazard management structures with an emphasis on flood and community-based disaster risk reduction are advisable.

Funding

This study (no: IR.MUK.REC.1398.223.) was conducted with the financial support of the Deputy for Health at Ministry of Health and Medical Education (MOHE) in IRAN. The funding body did not have any other roles in the study design, collection, analysis, and interpretation of data and in writing the manuscript.

Declaration of Competing Interest

The authors declare no potential conflict of interest.

Acknowledgements

This study was carried out with the support of the Social Determinants of Health Research Center, Research Institute for Health Development at Kurdistan University of Medical Sciences and Disaster Risk Management Office, Deputy for Health at Ministry of Health and Medical Education (MOHE) in IRAN. Moreover, the authors would like to thank all participants of the study who gave us their precious time.

References

1- IFRC, 2011: World Disasters Report 2011: International Federation of Red Cross and Red Crescent Societies. [Available online at https://media.ifrc.org/ifrc/wp-content/uploads/sites/5/2018/10/B-WDR-2018-EN-LR.pdf

2- Yari A, Ardalan A, Ostadtaghizadeh A, Zarezadeh Y, Boubakran MS, Bidarpoor F, Rahimiforoushani A. Underlying factors affecting death due to flood in Iran: A qualitative content analysis. International Journal of Disaster Risk Reduction. 2019 Nov 1;40:101258.https://doi.org/10.1016/j.ijdrr.2019.101258.

3- Yadollahie M. The flood in Iran: a consequence of the global warming?. The international journal of occupational and environmental medicine. 2019 Apr;10(2):54.doi: 10.15171/ijoem.2019.1681.

4- Ardalan A, Ostadtaghizadeh A, Sari A.B, et al., National Flood Report. Rescue, salvation and Health Working Group, Tehran, The Islamic Republic of Iran. final version (2019).

5- Becker JS, Taylor HL, Doody BJ, Wright KC, Gruntfest E, Webber D. A review of people's behavior in and around floodwater. Weather, Climate, and Society. 2015 Oct;7(4):321-32.https://doi.org/10.1175/WCAS-D-14-00030.1.

6- Jonkman SN, Kelman I. An analysis of the causes and circumstances of flood disaster deaths. Disasters. 2005 Mar;29(1):75-97.https://doi.org/10.1111/j.0361-3666.2005.00275.x.

7- Yari A, Ostadtaghizadeh A, Ardalan A, Zarezadeh Y, Rahimiforoushani A, Bidarpoor F. Risk factors of death from flood: Findings of a systematic review. Journal of environmental health science and engineering. 2020 Jul 24:1-1.https://doi.org/10.1007/s40201-020-00511-x.

8- Llewellyn M. Floods and Tsunamis. Surgical Clinics of North America. 2006;86(3):557-78. doi: 10.1016/j.suc.2006.02.006.

9- Keim ME. Floods. Koenig and schultz's Disaster Medicine: Comprehensive Principles and Practices2009. p. 529-42.

10- Di Mauro M, De Bruijn KM, Meloni M. Quantitative methods for estimating flood fatalities: towards the introduction of loss-of-life estimation in the assessment of flood risk. Natural hazards. 2012;63(2):1083-113. DOI: 10.1007/s11069-012-0207-4.

11- Pereira S, Zêzere JL, Quaresma I, Santos PP, Santos M. Mortality patterns of hydro-geomorphologic disasters. Risk Analysis. 2016 Jun;36(6):1188-210. DOI: 10.1111/risa.12516.

12- Jonkman SN. Loss of life caused by floods: an overview of mortality statistics for worldwide floods. DC1-233-6. 2003.http://resolver.tudelft.nl/uuid:2deb3bd4-bf22-45a7-be2f-c3ab269d52af.

13- Priest S. Building a model to estimate Risk to Life for European flood events. T10-0710. 2009. http://resolver.tudelft.nl/uuid:29529085-4933-4bf0-a124-7352c455daf0.

14- Kelman I. Philosophy of flood fatalities. Flood Risk Net Newsletter. 2004; 1:3-4.

15- Brons RK, Bierens JJLM. Water-Related Disasters. In: Bierens JJLM, editor. Handbook on Drowning: Prevention, Rescue, Treatment. Berlin, Heidelberg: Springer Berlin Heidelberg; 2006. p. 535-85.

16- World Health Organization (WHO) , Floods: climate change and adaptation strategies for human health. WHO, Regional Office for Europe, Geneva, Switzerland (2002).

17- Jonkman SN, Vrijling JK. Loss of life due to floods. Journal of Flood Risk Management.2008;1(1):43-56. https://doi.org/10.1111/j.1753-318X.2008.00006.x.
18- Ashley ST, Ashley WS. Flood fatalities in the United States. Journal of Applied Meteorology and Climatology. 2008;47(3):805-18. https://doi.org/10.1175/2007JAMC1611.1.
19- Egli T. Nonstructural flood plain management: measures and their effectiveness. International Commission for the Protection of the Rhine (ICPR); 2002.
20- Rae E, Campbell, P, Haynes, K, Gissing, A, Coates, L. Preventing flood related fatalities: a focus on people driving through floodwater. Non-peer reviewed research proceedings from the Bushfire and Natural Hazards CRC & AFAC conference. 30 August – 1 September 2016.
21- Grothmann T, Reusswig F. People at risk of flooding: why some residents take precautionary action while others do not. Natural hazards. 2006 May 1;38(1-2):101-20. https://doi.org/10.1007/s11069-005-8604-6.
22- Shabanikiya, H., Seyedin, H., Haghani, H. and Ebrahimian, A., 2014. Behavior of crossing flood on foot, associated risk factors and estimating a predictive model. Natural hazards, 73(2), pp.1119-1126.https://doi.org/10.1007/s11069-014-1124-5.
23- Lindell MK, Whitney DJ. Correlates of household seismic hazard adjustment adoption. Risk analysis. 2000 Feb;20(1):13-26.https://doi.org/10.1111/0272-4332.00002.
24- Miceli R, Sotgiu I, Settanni M. Disaster preparedness and perception of flood risk: A study in an alpine valley in Italy. Journal of environmental psychology. 2008 Jun 1;28(2):164-73.https://doi.org/10.1016/j.jenvp.2007.10.006.
25- Diakakis M, Priskos G, Skordoulis M. Public perception of flood risk in flash flood prone areas of Eastern Mediterranean: The case of Attica Region in Greece. International journal of disaster risk reduction. 2018 Jun 1;28:404-13. https://doi.org/10.1016/j.ijdrr.2018.03.018.
26- Ibrahim NF, Zardari NH, Shirazi SM, Mohd Haniffah MR, Mat Talib S, Yusop Z, Mohd Yusoff SM. Identification of vulnerable areas to floods in Kelantan River sub-basins by using flood vulnerability index. International Journal of GEOMATE. 2017 Jan 1;12(29):107-14.DOI:10.21660/2017.29.11110.
27- Sharif HO, Jackson TL, Hossain MM, Zane D. Analysis of flood fatalities in Texas. Natural Hazards Review. 2015 Feb 1;16(1):04014016.https://doi.org/10.1061/(ASCE)NH.1527-6996.0000145.
28- Salvati P, Petrucci O, Rossi M, Bianchi C, Pasqua AA, Guzzetti F. Gender, age and circumstances analysis of flood and landslide fatalities in Italy. The Science of the total environment. 2018;610-611:867-79. https://doi.org/10.1016/j.scitotenv.2017.08.064.
29- Haynes K, Coates L, van den Honert R, Gissing A, Bird D, de Oliveira FD, et al. Exploring the circumstances surrounding flood fatalities in Australia-1900-2015 and the implications for policy and practice. Environmental Science & Policy. 2017; 76:165-76. https://doi.org/10.1016/j.envsci.2017.07.003.
30- Kundzewicz Z, Kundzewicz W. Mortality in flood disasters. Extreme weather events and public health responses: Springer; 2005. p. 197-206. https://doi.org/10.1007/3-540-28862-7_19.

31- Lee, S. and K. Vink, Assessing the vulnerability of different age groups regarding flood fatalities: case study in the Philippines. Water Policy, 2015. 17(6): p. .1061-1045. https://doi.org/10.2166/wp.2015.089.

32- Jongman B, Winsemius HC, Aerts JC, De Perez EC, Van Aalst MK, Kron W, Ward PJ. Declining vulnerability to river floods and the global benefits of adaptation. Proceedings of the National Academy of Sciences. 2015 May 5;112(18):E2271-80.doi: 10.1073/pnas.1414439112

33- Yari A, Ardalan A, Ostadtaghizadeh A, Rahimiforoushani A, Zarezadeh Y, Bidarpoor F, investigating the factors Affecting Death Due to Flood in Iran. Doctorate Thesis in Tehran University of medical sciences (2019).

34- Netemeyer RG, Bearden WO, Sharma S. Scaling procedures: Issues and applications. London: Sage; 2003.

35- Pourhoseingholi MA, Baghestani AR, Vahedi M. How to control confounding effects by statistical analysis. Gastroenterology and hepatology from bed to bench. 2012;5(2):79.

36- Lowe D, Ebi K, Forsberg B. Factors increasing vulnerability to health effects before, during and after floods. International journal of environmental research and public health.2013;10(12):7015-67. https://doi.org/10.3390/ijerph10127015.

37- Paul, B.K. and S. Mahmood, Selected physical parameters as determinants of flood fatalities in Bangladesh, 1972–2013. Natural Hazards, 2016. 83(3): p. 1703-1715. https://doi.org/10.1007/s11069-016-2384-z.

38- FitzGerald G, Du W, Jamal A, Clark M, Hou XY. Flood fatalities in contemporary Australia (1997–2008). Emergency Medicine Australasia. 2010;22(2):180-6. https://doi.org/10.1111/j.1742-6723.2010.01284.x.

39- Terpstra T, Gutteling JM. Households' perceived responsibilities in flood risk management in the Netherlands. International Journal of Water Resources Development.2008;24(4):555-65. https://doi.org/10.1080/07900620801923385.

40- Samir KC. Community Vulnerability to Floods and Landslides in Nepal. Ecology and Society. 2013;18(1):8. http://dx.doi.org/10.5751/ES-05095-180108.

41- Shaw R, Shiwaku Hirohide Kobayashi K, Kobayashi M. Linking experience, education, perception and earthquake preparedness. Disaster Prevention and Management: An International Journal. 2004;13(1):39-49. https://doi.org/10.1108/09653560410521689.

42- King D. You're on your own: Community vulnerability and the need for awareness and education for predicatable natural disasters. Journal of contingencies and crisis management. 2000;8(4):223-8. https://doi.org/10.1111/1468-5973.00143.

43- Benadusi M. Pedagogies of the unknown: Unpacking 'culture'in disaster risk reduction education. Journal of Contingencies and Crisis Management. 2014;22(3):174-83. https://doi.org/10.1111/1468-5973.12050.

44- Jackson TL. The impacts of increasing rainfall: Flood fatalities in Texas: The University of Texas at San Antonio; 2009.

45- Duclos P, Vidonne O, Beuf P, Perray P, Stoebner A. Flash flood disaster-nîmes, France, 1988. European Journal of Epidemiology. 1991;7(4):365-71. https://doi.org/10.1007/BF00145001.

46- Lumbroso D, Vinet F. A comparison of the causes, effects and aftermaths of the coastal flooding of England in 1953 and France in 2010. Natural Hazards and Earth System Sciences. 2011; 11:2321-33.https://doi.org/10.5194/nhess-11-2321-2011.
47- Brazdova M, Riha J. A simple model for the estimation of the number of fatalities due to floods in central Europe. Natural Hazards and Earth System Sciences. 2014;14(7):1663-76. https://doi.org/10.5194/nhess-14-1663-2014
48- Sharif HO, Jackson T, Hossain M, Bin-Shafique S, Zane D. Motor vehicle-related flood fatalities in Texas, 1959–2008. Journal of Transportation Safety & Security.2010;2(4):325-35. https://doi.org/10.1080/19439962.2010.506596.
49- Coates L. Flood fatalities in Australia, 1788-1996. Australian Geographer.1999;30(3):391-408. https://doi.org/10.1080/00049189993657.
50- Priest SJ, Wilson T, Tapsell SM, Penning-Rowsell EC, Viavattene C, Fernandez-Bilbao A. Building a model to estimate risk to life for European flood events–final report. 2007. https://eprints.mdx.ac.uk/id/eprint/16266.
51- Vinet F, Lumbroso D, Defossez S, Boissier L. A comparative analysis of the loss of life during two recent floods in France: the sea surge caused by the storm Xynthia and the flash flood in Var. Natural hazards. 2012;61(3):1179-201.https://doi.org/10.1007/s11069-011-9975-5.
52- Benadusi M. Pedagogies of the unknown: Unpacking 'culture'in disaster risk reduction education. Journal of Contingencies and Crisis Management. 2014;22(3):174-83. https://doi.org/10.1111/1468-5973.12050.
53- Wallingford H. Flood Risks to People Phase 2. Final Report Prepared for Defra/Environment Agency Flood and Coastal Defence R&D Programme. 2006.
54- Thieken AH, Kreibich H, Müller M, Merz B. Coping with floods: preparedness, response and recovery of flood-affected residents in Germany in 2002. Hydrological Sciences Journal. 2007;52(5):1016-37. https://doi.org/10.1623/hysj.52.5.1016.

Figure

Click here to access/download;Figure;figure.pdf

Figure 1: Flood death Rate per 10,000,000 in Iran:2019.

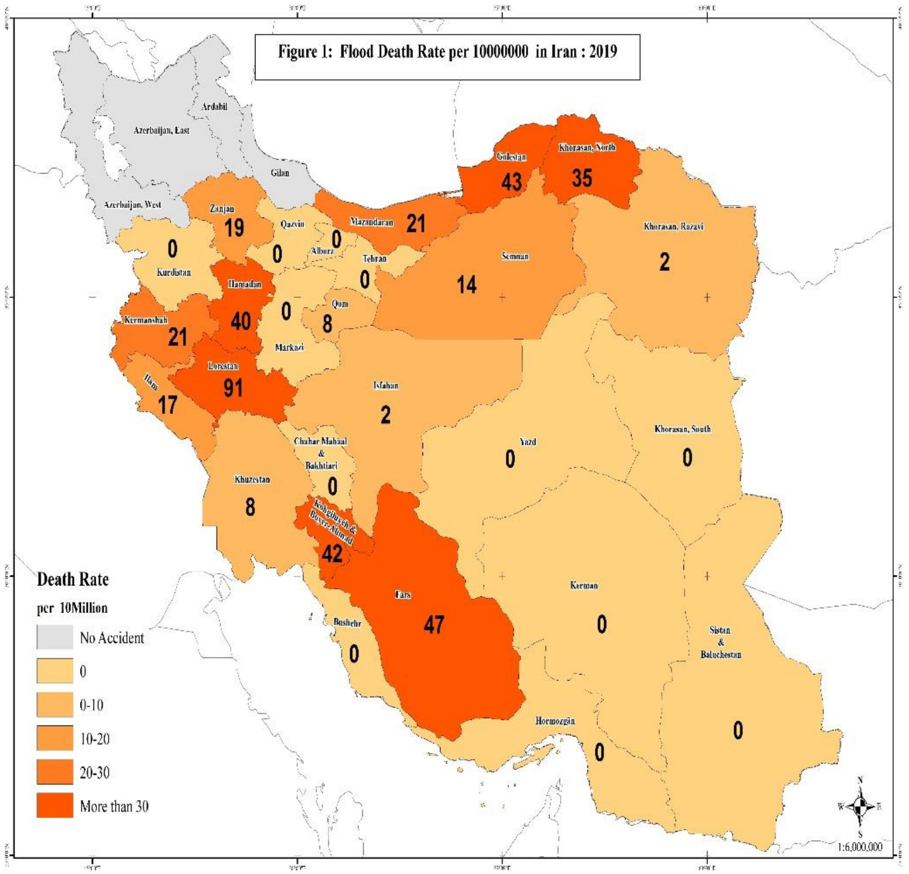

Figure 2: Flood injury Rate per 10,000,000 in Iran:2019.

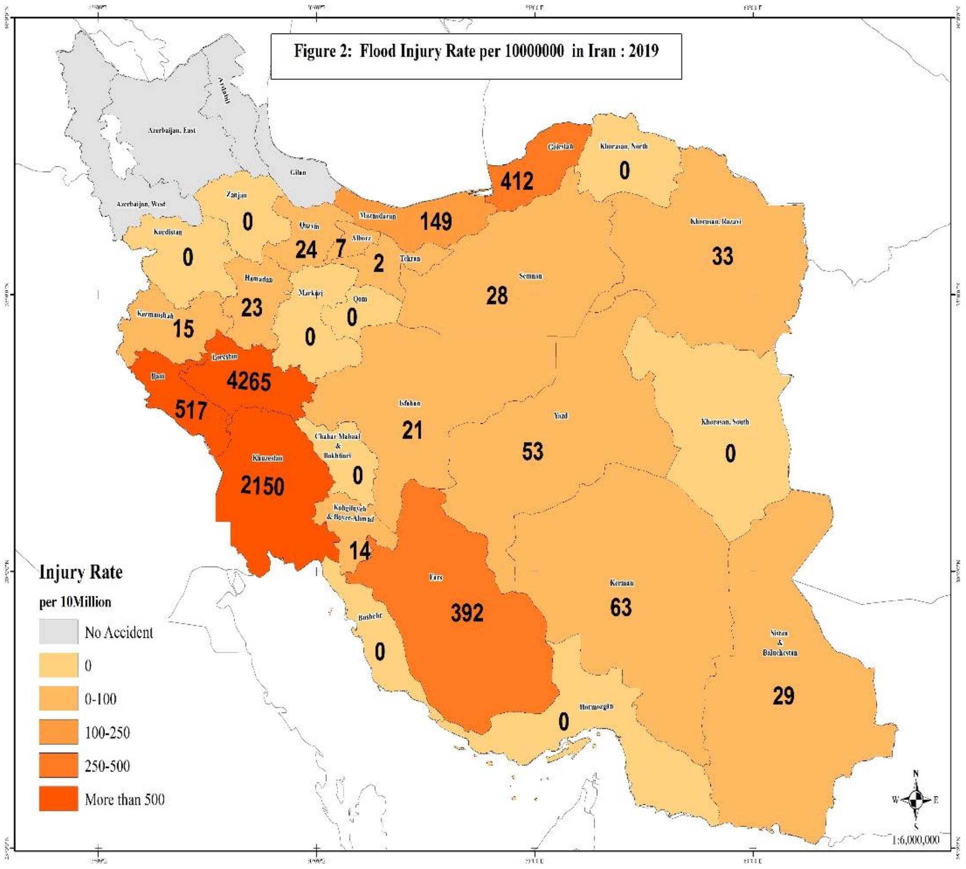

These maps were constructed by the authors using existing data in various sources in Iran's floods in 2019.
